# Alternative splicing of BAZ1A in colorectal cancer disrupts the DNA damage response and increases chemosensitization

**DOI:** 10.1038/s41419-024-06954-6

**Published:** 2024-08-07

**Authors:** Nivedhitha Mohan, Gavin S. Johnson, Jorge Enrique Tovar Perez, Wan Mohaiza Dashwood, Praveen Rajendran, Roderick H. Dashwood

**Affiliations:** 1 Center for Epigenetics & Disease Prevention, Texas A&M Health, Houston, TX USA; 2 Department of Translational Medical Sciences, Texas A&M College of Medicine, Houston, TX USA; 3 Present Address: CRISPR Therapeutics, South Boston, MA USA

**Keywords:** Cancer prevention, Colorectal cancer, Colon cancer

## Abstract

Bromodomain Adjacent to Zinc Finger Domain 1A (BAZ1A) is a critical regulator of chromatin remodeling. We sought to clarify the roles of BAZ1A in the etiology of colorectal cancer, including the mechanisms of its alternatively spliced variants. Public databases were examined and revealed high BAZ1A expression in the majority of colorectal cancer patients, which was corroborated in a panel of human colon cancer cell lines. BAZ1A silencing reduced cell viability and increased markers of DNA damage, apoptosis, and senescence, along with the downregulation of Wnt/β-catenin signaling. The corresponding molecular changes resulted in tumor growth inhibition when BAZ1A-knockout cells were implanted into nude mice. In rescue experiments, a short isoform of BAZ1A that was associated with alternative splicing by the DBIRD complex failed to restore DNA repair activity in colon cancer cells and maintained chemosensitivity to phleomycin treatment, unlike the full-length BAZ1A. A working model proposes that a buried domain in the N-terminus of the BAZ1A short isoform lacks the ability to access linker DNA, thereby disrupting the activity of the associated chromatin remodeling complexes. Given the current interest in RNA splicing deregulation and cancer etiology, additional mechanistic studies are warranted with new lead compounds targeting BAZ1A, and other members of the BAZ family, with a view to improved therapeutic interventions.

## Introduction

Cancer etiology is characterized by the deregulation of epigenetic signatures, including altered DNA methylation states, noncoding RNAs, and post-translational modifications to histone and nonhistone proteins that affect chromatin condensation and DNA accessibility [1–4]. Chromatin remodeling factors exert a wide range of biological functions via direct and indirect effects on DNA packaging and gene expression [4]. Bromodomain Adjacent to Zinc Finger Domain 1A (BAZ1A) is a non-catalytic subunit of the ATP-dependent chromatin assembly factor that is involved in chromatin assembly, DNA replication, DNA repair, and transcriptional regulation [5–12]. BAZ1A enhances chromatin compaction, regulates nucleosome spacing together with the protein partner SWI/SNF Related, Matrix Associated, Actin Dependent Regulator of Chromatin Subfamily A Member 5 (SMARCA5), and plays a vital role in DNA replication in densely-packed chromatin regions [6, 13, 14]. Loss of either BAZ1A or SMARCA5 depletes their colocalization in replicating pericentromeric heterochromatin and leads to a delay in cell-cycle progression during S phase [6]. A BAZ1A mutant that cannot interact with SMARCA5 interferes with replication in condensed chromatin, highlighting the critical role of the BAZ1A-SMARCA5 complex in DNA replication processes at densely packed chromatin regions [6].

These proteins also are crucial for efficient DNA repair [9, 10]. During nucleotide excision repair, BAZ1A is recruited by the histone methyltransferase Mixed Lineage Leukemia Protein-1 (MLL1) following the methylation of histone H3K4, thereby aiding in the removal of pyrimidine dimers [11]. Depletion of BAZ1A or SMARCA5 leads to double-strand break sensitivity and DNA damage, affecting both non-homologous end joining (NHEJ) and homologous recombination (HR) [9, 15, 16]. Notably, Baz1a knockout mice are viable and are capable of repairing DNA double-strand breaks in vivo [17], presumably due to compensatory actions of other Baz family members akin to the human counterparts BAZ1B, BAZ2A or BAZ2B. Highlighting critical roles beyond chromatin compaction, the bromodomain (BRD) in BAZ1A binds relatively weakly to acetylated histone peptides [18], making it more available to interact with acetylated non-histone proteins such as Cell Cycle and Apoptosis Regulator (CCAR2) – a Wnt coactivator in colon cancer cells (see below). Genetically engineered BRD or Plant Homeodomain (PHD) mutants revealed that ‘reader’ modules in BAZ1A and BAZ1B are required for DNA damage recovery [18]. In particular, BAZ1A was found to be essential for the loading of Imitation SWItch (ISWI) factors at DNA lesions [18]. These observations provide insight into the broader roles of BAZ1A beyond histone binding and chromatin compaction, encompassing additional key regulatory involvement in DNA repair and cell signaling pathways.

Knockdown (KD) of BAZ1A triggered senescence-associated phenotypes in various human cancer cell lines due to increased SMAD Family Member 3 (SMAD3) expression promoting p21 activation [19]. Upregulation of BAZ1A in HER2+ breast cancer patients was associated with poor overall survival [20], and mutation of BAZ1A was documented in uterine carcinosarcoma [21]. In African American prostate cancer cohorts, BAZ1A expression was reduced and affected vitamin D receptor-dependent gene expression and disease progression [22]. In colon cancer cells treated with the deacetylase inhibitor sulforaphane (SFN) [23–26], acetylation of CCAR2 in the C-terminus established an acetyl ‘switch’ site [27], whereas acetylation in the N-terminus provided recognition motifs for the bromodomains of BAZ1A and ASH1-like protein (ASH1L). These acetyl reader interactions displaced β-catenin from the N-terminus of CCAR2 and lowered CCAR2/β-catenin associations, thereby interfering with the Wnt coactivator role of CCAR2 [28–31].

Another unreported finding in SFN-treated human colon cancer cells was the alternative splicing of *BAZ1A*, extending prior observations on exon skipping/inclusion in a mouse model of prostate cancer after feeding SFN in the diet [32]. The present investigation sought to clarify the functional consequences of *BAZ1A* alternative splicing in human colon cancer cells in the regulation of DNA damage response. The alternatively spliced short isoform of BAZ1A (BAZ1A-SF, short-form) was found to be markedly less effective than the full-length isoform (BAZ1A-LF, long-form) in DNA damage repair. Computational modeling suggested that the different conformation of BAZ1A-SF affected interactions with the key protein partner SMARCA5, potentially burying a critical DNA interaction domain, although this awaits further mechanistic corroboration.

## Materials and methods

### Cells and treatments

Non-transformed CCD841 human colonic epithelial cells and colon and duodenal cancer HCT116, SW480, SW620, HT29, Caco2, HUTU80, LOVO, and SW48 cells were used within 10–15 passages following receipt from ATCC (Manassas, VA). As reported [33], each cell line was independently verified to be of human origin, free from interspecies contamination, and possessing the correct genetic profile based on allele-specific markers (Idexx Radil, Columbia, MO). Cells were cultured in McCoy’s 5A or Eagle’s Minimum Essential Media (Invitrogen) supplemented with 10% fetal bovine serum and 1% penicillin/streptomycin and maintained at 37 °C in a humidified chamber with 5% CO_2_. Test agents, including SFN, 6-methylsulfinylhexyl isothiocyanate (6-SFN), Phleomycin (ThermoFisher), BG45 and Tubacin (MedChem Express) were incubated at concentrations indicated in the figures, with dimethylsulfoxide (DMSO) as vehicle control.

### *BAZ1A* knockdown

Three shRNAs targeting different regions of *BAZ1A*, i.e., shRNA#1 and shRNA#2 targeting the coding sequence and shRNA#3 targeting the 3′-UTR (Sigma-Aldrich, St. Louis, MO), were packaged into lentiviral particles and purified using Lenti-X concentrator (Takara Bio, San Jose, CA). Colon cancer cells HCT116 and SW620 were stably transduced using Polybrene reagent (Sigma-Aldrich). Cells were initially treated with puromycin concentrations in the range 2–5 μg/ml for 48–72 h, and stable clones were propagated with puromycin maintenance concentrations in the range 0.1–0.5 μg/ml (Sigma-Aldrich). For sequences of shRNAs refer to Supplementary Table 1.

### Site-directed mutagenesis

GFP-BAZ1A (Plasmid # 65371) was obtained from Addgene. Site-directed mutagenesis, PCR amplification and sub-cloning into pcDNA6.2/N-EmGFP-DEST generated BAZ1A-LF and BAZ1A-SF constructs, with exon-13 excision confirmed by sequencing. Constructs were transiently transfected into BAZ1A stable knockout cells and lysates were harvested after 48 h for molecular analysis.

### Immunoblotting

Immunoblotting (IB) procedures followed published methodologies for whole cell lysate and tissue lysate from tumor xenografts [27, 34–38]. Primary antibodies to BAZ1A and BAZ1B were from Bethyl Labs (Montgomery, TX), whereas BAZ2A and SMARCA5 antibodies were from Abcam (Waltham, MA) and Thermo Fisher (Waltham, MA), respectively. Antibodies to p16 and hnRNPA1 were from Santa Cruz Biotechnology (Dallas, TX), and those to histones H3, H4, H3K27ac, H3K27me3, H3K9ac, H3K14ac, phosphorylated MYC(Ser), and β-galactosidase were from Cell Signaling Technology (Danvers, MA). The antibody to H2A was from Active Motif (Carlsbad, CA). Antibody to ZIRD (ZNF326) was generated by the Antibody and Biopharmaceuticals core facility in the Center for Epigenetics & Disease Prevention at Texas A&M Health (Houston, TX). pH2AX, poly(ADPribose)polymerase (PARP), β-catenin, c-Myc, CCAR2, matrix metalloproteinase 7 (MMP7), p53, p21, Caspase-3 and β-actin primary antibodies were from sources reported previously [25–27].

### Clonogenicity assays

The basic methodologies for cell viability assays were as reported [39]. Briefly, cells in the exponential growth phase were plated in 96-well tissue culture plates and after attachment overnight, they were treated for 48 h in triplicate wells and assessed using the Cell Counting Kit-8 (CCK8, ApexBio, Boston, MA, USA). Alternatively, cells were plated in 6-well dishes (500 cells/well), allowed to attach overnight, and then treated for 48 h in triplicate. Seven days later, colonies were fixed, stained with crystal violet, and counted. The surviving fraction was calculated as the ratio of the number of colonies in the treated sample to the number of colonies in the vehicle control.

### Apoptosis assays

Cells were treated for 48 h in triplicate and assessed using the Pharmingen PE Annexin V Apoptosis Detection Kit I (BD Biosciences, San Jose, CA, USA). Briefly, cells were collected, washed with PBS, and incubated in binding buffer with 5 μl of PE Annexin V for 5 min and 5 μl of 7-AAD for 15 min in the dark at 37 °C. The percent of apoptotic cells was determined using LSR II Flow cytometer (BD Biosciences) and FlowJo 10.8.1 software.

### Reporter assays

BAZ1A KD cells (see above) were transiently transfected with 1 μg of plasmid DNA for the Wnt reporter construct TOPflash, or the negative control FOPflash (Upstate Biotechnology, Lake Placid, NY, USA). Firefly luciferase activity was corrected for Renilla luciferase activity to control for transfection efficiency, as reported [23, 36].

### RNA analyses

Reverse transcription quantitative real-time PCR (RT-qPCR) analysis of *BAZ1A* and other target genes followed the methodologies reported [25, 27], with *Glyceraldehyde-3-Phosphate Dehydrogenase* (*GAPDH*) as the nominal loading control. For sequences of PCR primers used in this investigation refer to Supplementary Table 2.

### Docking in silico

BAZ1A-LF and BAZ1A-SF structures were predicted using AlphaFold2 (EMBL-EBI, Hinxton, UK) and visualized using PyMol v.2.5.4. After pairwise alignment, docking of SMARCA5 to BAZ1A-LF and BAZ1A-SF structures was performed using HDOCK v.1.1, and protein-protein interactions were visualized using PyMOL v.2.5.4.

### Chromatin immunoprecipitation

The ChIP-IT Express Enzymatic kit (Active Motif, Carlsbad, CA) was used for chromatin immunoprecipitation (ChIP). Following a reported methodology [27, 34], CCAR2 knockout (CCAR2^-/-^) and parental HCT116 cells were cross-linked with formaldehyde and homogenized to isolate the nuclear fraction. DNA fragmentation was performed by enzymatic digestion for 15 cycles of 20 s each. Ten microliters of fragmented chromatin were used as input control and the remainder was subjected to IP with anti-BAZ1A antibody (Bethyl labs). After reversing the cross-linking and proteinase treatment, DNA was purified using the QIAquick PCR Purification kit (Qiagen, Hilden, Germany). PCR was run on BioRad Light Cycler 480 II with pre-incubation for 5 min at 95 °C, then 55 cycles at 95 °C for 10 s, 60 °C for 10 s, and 72 °C for 10 s. Primer sequences for interrogating the c-*MYC* gene were as reported [27].

### Immunofluorescence assays

Cells were grown on glass coverslips pre-coated with poly-L-Lysine (Sigma, #P1399). Following treatment with test agent, vehicle, DNA construct or vector control, cells were fixed with 2% phosphate buffered formalin for 10 min and permeabilized with 2.1% citric acid and 0.5% Tween 20 in distilled water (pH 2.0) for 10 min at room temperature. Samples were blocked in 1% BSA and incubated overnight with primary antibody to pH2AX Ser139 (#9718, Cell Signaling), BAZ1A (#A301-318A, Bethyl labs), β-galactosidase (#27198, Cell Signaling) or H3 (#39064, Active Motif) followed by secondary antibody coupled to AlexaFluor 555 (#A32732, 1:250, ThermoFisher) for 1 h. 4′,6-Diamidino-2-phenylindole (DAPI) was used as counterstain for nuclei (Prolong Gold antifade reagent, Molecular Probes). Fluorescent images were captured on a Confocal microscope (W1-Yokogawa-Ti1-Nikon spinning disk) for image acquisition and analyzed using Image J.

### Senescence assays

Cells seeded and cultured for 24 h, as above, were fixed with 4% paraformaldehyde. After fixation, cells were stained and analyzed using the CellEvent^TM^ Senescence Green Detection Kit following the manufacturer’s recommended protocol (ThermoFisher). The fluorescence signal was captured using a Cytation 5 microplate reader (BioTek).

### In vivo studies

Preclinical experiments received prior approval from the Institutional Animal Care and Use Committee. Following a reported methodology for human colon cancer cells [27, 38], 5 × 10^6^ *BAZ1A* knockout or vector control cells were injected into either flank of male athymic nude mice, with n = 5 animals per group. Body weights and tumor growth were assessed twice a week. Tumor volumes were measured after 30 days using calipers.

### Statistics

For all the experimental procedures outlined above, results shown in the figures are representative findings from at least three biological and three technical replicates, with data expressed as mean ± SE, unless indicated otherwise. Student’s t-test was used for paired comparisons, whereas multiple groups were subjected to analysis of variance (ANOVA) and Bonferroni’s test (GraphPad Prism™ v5.04, La Jolla, CA, USA). Statistical significance in the figures was shown as *P < 0.05, **P < 0.01, ***P < 0.001, or ****P < 0.0001.

## Results

### BAZ1A is overexpressed in human colorectal cancer

**Fig. 1:**
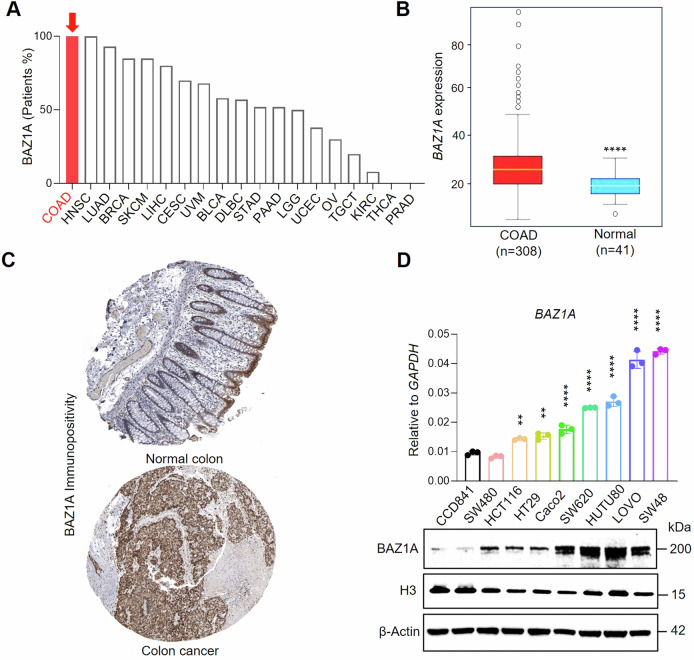
BAZ1A overexpression in human colorectal cancer. **A** BAZ1A protein expression determined by immunohistochemical analysis; colon adenocarcinoma (COAD), red bar. **B** *BAZ1A* transcript levels in COAD versus normal colon tissue. **C** Comparative BAZ1A protein expression in human tissue microarrays. **D** *BAZ1A* transcript expression by RT-qPCR and BAZ1A protein levels by immunoblotting across various colon and duodenal cancer cell lines, compared to the normal colonic epithelial cell line CCD841. Data from The Human Protein Atlas and The Cancer Genome Atlas (TCGA). Statistical significance was denoted by **p < 0.01 and ****p < 0.0001.

Public databases revealed BAZ1A expression in human colon adenocarcinoma (COAD) and other cancer types. Notably, almost 100% of colorectal cancer patients had BAZ1A detectable in tumor samples (Fig. 1A, red bar), with high BAZ1A immunopositivity in colon cancer tissue microarrays compared with normal colon samples (Fig. 1B). *BAZ1A* mRNA expression also was significantly higher in COAD versus normal tissues (Fig. 1C). In a panel of colon and duodenal cancer cell lines (Fig. 1D), RT-qPCR analyses revealed significantly increased constitutive mRNA expression of *BAZ1A* compared to a normal colonic epithelial cell line (CCD841), with the exception of SW480 cells, and IB revealed concordance at the protein level, using histone H3 and β-actin as loading controls.

### BAZ1A knockdown in colon cancer cells decreases cell viability while increasing DNA damage, apoptosis, and senescence markers

**Fig. 2:**
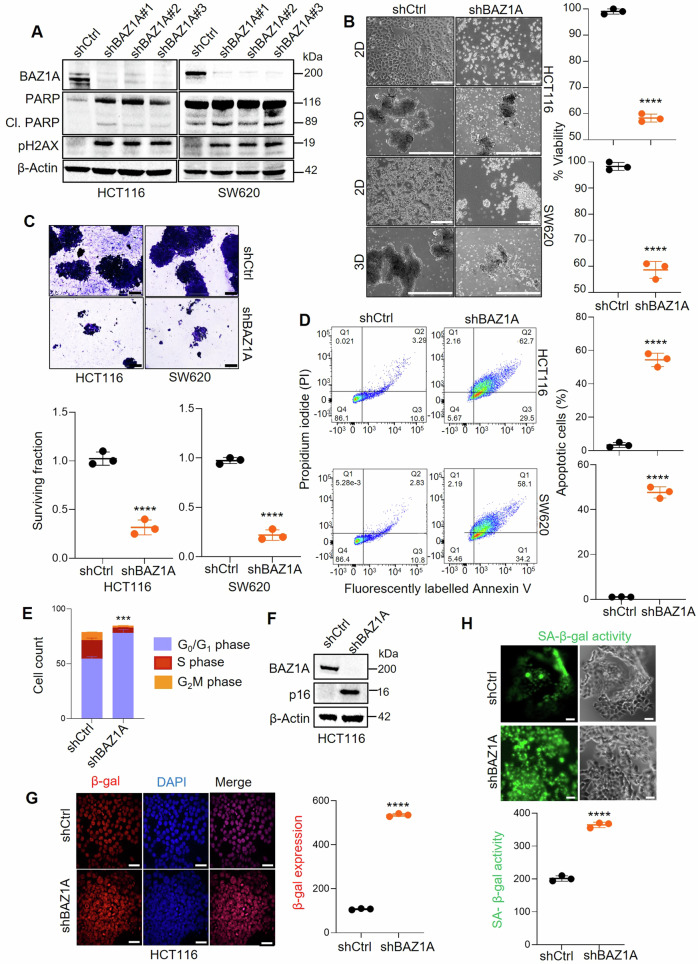
Genetic knockdown of BAZ1A affects viability, apoptosis, DNA damage, and senescence in human colon cancer cells. **A** Immunoblotting in HCT116 and SW620 cells after stable transfection of scrambled shRNA control (shCtrl) or three different shRNAs targeting BAZ1A (shBAZ1A #1, #2, #3), with β-Actin as loading control. **B** Two-dimensional (2D) and three-dimensional (3D) morphology, and associated cell viability in the CCK8 assay; scale bar = 200 μm. **C** Representative images (×4 magnification) from colony-formation assay and quantification of crystal-violet-stained colonies. **D** Fluorescence-activated cell sorting (FACS) and quantification of apoptosis in stable BAZ1A KD cells. **E** FACS-based quantification of cell count of shCtrl and shBAZ1A cells at different stages of cell cycle. **F** Immunoblotting in HCT116 shCtrl and shBAZ1A KD cells with β-Actin as loading control. **G** Immunofluorescence detection of β-galactosidase (β-gal) in shCtrl and shBAZ1A KD cells and quantification of pixel intensity; scale bar = 17 μm. **H** Senescence associated β-galactosidase activity in shCtrl and shBAZ1A KD cells and quantification of pixel intensities. Differences between means were assessed using Student’s t-test for n = 3 replicates, indicated by ****p < 0.0001 vs. shCtrl.

In HCT116 and metastasis-lineage SW620 colon cancer cell lines [38], shRNA-mediated KD of BAZ1A induced PARP cleavage and pH2AX protein levels markedly, indicative of apoptosis and increased DNA damage, respectively (Fig. 2A). Morphological examination by microscopy along with quantification in the CCK8 assay revealed that BAZ1A silencing significantly reduced cell viability (Fig. 2B), and the colony-formation assay corroborated a decrease in the number of crystal violet-stained cells (Fig. 2C). In addition, fluorescence-activated cell sorting analyses identified a significant increase in the percentage of apoptotic cells following BAZ1A KD (Fig. 2D), coinciding with a greater proportion of cells having entered G_0_/G_1_ of the cell cycle and fewer cells in G_2_M and S phase (Fig. 2E). Consistent with previous findings [19], BAZ1A KD led to senescence-associated phenotypes, based on morphological examination (Fig. 2B) and molecular markers such as p16 induction (Fig. 2F) and increased β-galactosidase (β-gal) protein expression (Fig. 2G), indicating lysosomal β-gal content as a hallmark of senescent cells [40, 41]. Senescence-associated β-gal (SA-β-gal) activity was also significantly elevated in BAZ1A knockout cells compared to vector controls, as shown by the intensity of fluorescence staining and corresponding phase contrast images (Fig. 2H).

### BAZ1A knockdown attenuates Wnt/β-catenin signaling in colon cancer cells

**Fig. 3:**
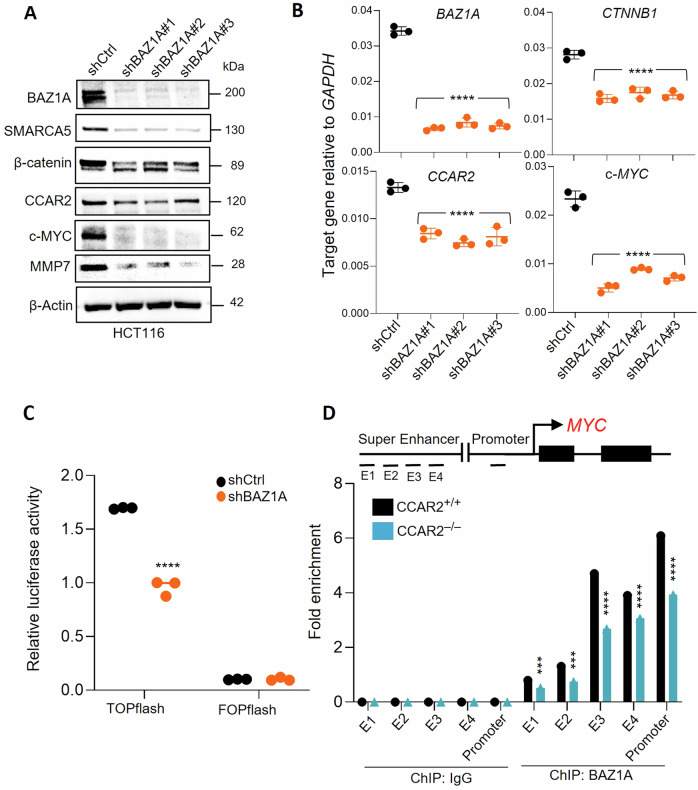
BAZ1A knockdown downregulates the Wnt/β-catenin pathway in colon cancer cells. **A** Immunoblotting in HCT116 cells after stable transfection with scrambled shCtrl or three different shRNAs targeting BAZ1A, with β-Actin as loading control. **B** RT-qPCR analysis of *BAZ1A*, *CTNNB1*, *CCAR2*, and c-*MYC* in shCtrl and shBAZ1A cells. **C** shCtrl and shBAZ1A KD cells were transiently transfected with TCF/LEF-responsive luciferase reporter construct TOPFlash or negative control FOPFlash vectors, along with Renilla pRL-TK plasmid to correct for transfection efficiency. **D** BAZ1A interactions on c-*MYC* were examined using ChIP assays in parental HCT116 CCAR2^+/+^ or HCT116 CCAR2^-/-^ cells, with IgG serving as negative control. Statistical significance determined by Student’s t-test for n = 3 replicates, indicated by ***p < 0.001, ****p < 0.0001 vs. shCtrl.

In BAZ1A knockout cells, IB revealed marked downregulation of the chromatin remodeling partner of BAZ1A, SMARCA5, as well as multiple components of the Wnt/β-catenin signaling pathway [27], including β-catenin, CCAR2, MMP7 and c-MYC (Fig. 3A). The latter proteins were examined because the loss of SMARCA5 expression in the context of BAZ1A KD (Fig. 3A) would be synonymous with reduced cell proliferation and decreased nuclear β-catenin [29, 42]. Significant downregulation of c-*MYC* and other Wnt/β-catenin genes was corroborated in RT-qPCR experiments (Fig. 3B). These findings in HCT116 cells were recapitulated in SW620 cells at the RNA and protein level (Fig. S1A, B, respectively). Using a Wnt reporter assay [23, 36], BAZ1A KD reduced TOPflash-associated luciferase activity compared to control, whereas no change was observed with the negative control reporter FOPflash (Fig. 3C). Chromatin-associated interactions have been identified in cells transfected with TOPflash and FOPflash [23], but corroborative evidence was sought via an endogenous β-catenin/Tcf target gene. Using a published ChIP methodology [27], the presence of BAZ1A was confirmed at both promoter and enhancer regions of c-*MYC* – interactions that were reduced significantly in CCAR2-null cells (Fig. 3D). The partial rather than complete loss of BAZ1A on promoter and enhancer regions is consistent with Wnt coactivators, other than CCAR2, also regulating c-*MYC* expression. Further studies are warranted to identify the transcriptional coactivator(s) and their influence on BAZ1A-regulated target genes such as c-*MYC*.

### BAZ1A knockdown suppresses tumor growth and alters histone marks in vivo

**Fig. 4:**
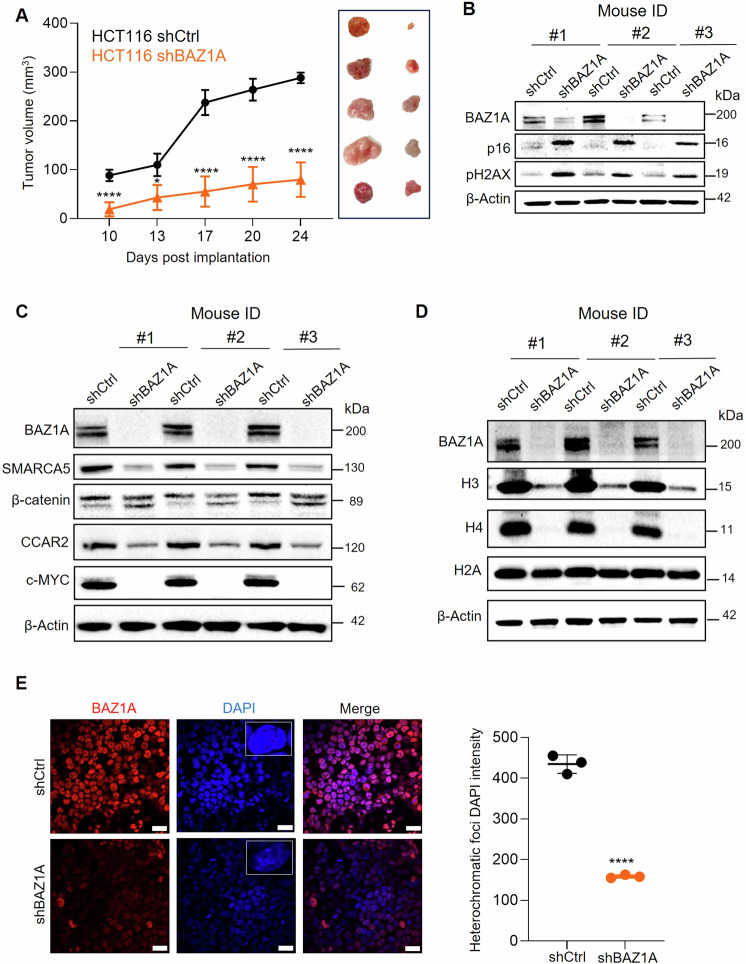
BAZ1A knockdown reduces tumor volume, induces DNA damage and senescence, downregulates Wnt/β-catenin pathway, and alters core histones. **A** Tumor volume over time in nude mice implanted with HCT116 cells expressing control shRNA (shCtrl) or BAZ1A shRNA (shBAZ1A), with inset images showing harvested tumors at the end of the study. Each data-point represents mean ± SD (n = 5). **B**–**D** Immunoblot analysis in shBAZ1A KD compared to shCtrl xenografts, with β-Actin as loading control (n = 3 mice). **E** Immunofluorescence of BAZ1A and DAPI staining of heterochromatin regions in the nuclei (inset) of shBAZ1A KD and shCtrl cells. Image analysis using ImageJ, with normalized brightness and contrast settings; scale bar = 17 μm. Statistical analysis showed significant differences, indicated by *p < 0.05 and ****p < 0.0001, as determined by Student’s t-test vs. shCtrl.

In a well-characterized xenograft model using immunodeficient mice, the growth of implanted BAZ1A knockout colon cancer cells was reduced compared to the control group, as evidenced by the significantly smaller tumor volumes over time (Fig. 4A). Immunoblotting of tumor xenografts recovered at the end of the experiment confirmed the effective KD of BAZ1A and the corresponding increase in DNA damage and senescence markers, such as pH2AX and p16 (Fig. 4B). Additionally, the downregulation of SMARCA5 and Wnt/β-catenin signaling proteins, including c-MYC, was observed in the BAZ1A knockout xenografts (Fig. 4C). Using histone H2A as a loading control, a marked depletion of histone H3 and histone H4 protein expression was observed in BAZ1A-null xenografts (Fig. 4D). We corroborated the reduced expression of H3 and H4 core histone proteins in BAZ1A KD cell lines by immunofluorescence (Fig. S2A) and IB (Fig. S2B). Notably, the proteasome inhibitor MG132 restored both H3 and H4 levels in BAZ1A KD cells (Fig. S2C), consistent with prior findings indicating that DNA damage led to histone degradation mediated by the proteasome machinery [43]. The cell cycle arrest observed in BAZ1A KD cells (Fig. 2E) is likely due, in part, to decreased deposition of newly synthesized core histones during S phase [44]. Thus, rather than regulating histone post-translational modifications, BAZ1A KD appeared to act upstream, at the nucleosome level. Consistent with this notion, heterochromatic foci were reduced significantly in BAZ1A-null tumor xenografts recovered at the end of the study (Fig. 4E).

### *BAZ1A* alternative splicing influences DNA damage in colon cancer cells

**Fig. 5:**
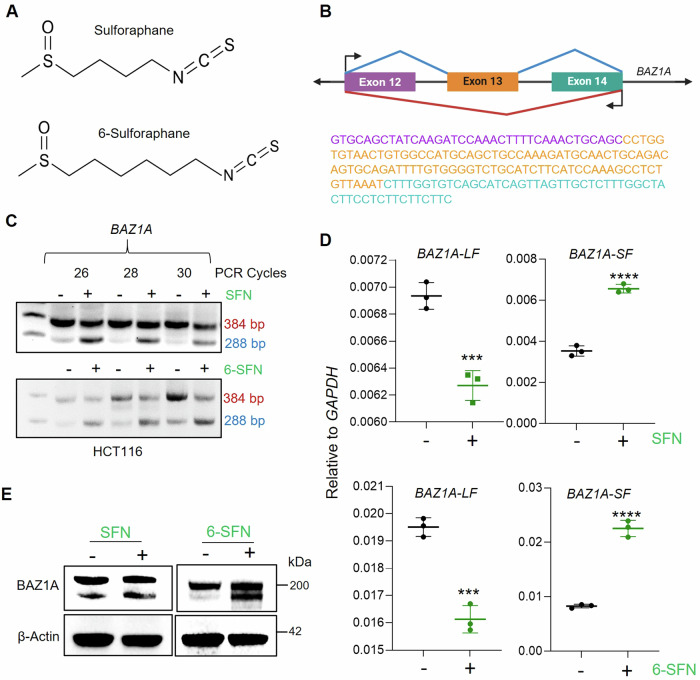
Sulforaphane and 6-sulforaphane promote BAZ1A alternative splicing in colon cancer cells. **A** Chemical structure of sulforaphane (SFN) and 6-sulforaphane (6-SFN). **B** Schematic illustration of *BAZ1A* exon-13 skipping, depicting the full-length (red line) and the alternatively spliced form (blue line) excluding exon-13 (orange box). Sanger sequencing results, displaying exon 13 (orange) flanked by partial sequences of exons 12 (purple) and 14 (blue), illustrating the splice sites. **C** RT-PCR analysis of the *BAZ1A* transcript region spanning exons 12–14, conducted over 26–30 PCR cycles in HCT116 cells treated with 15 µM of SFN or 6-SFN for 6 h. The analysis distinguished between long (384 bp) and short forms (288 bp) of the transcript. **D** RT-qPCR quantification of *BAZ1A* long form (LF) and short form (SF) transcripts upon SFN or 6-SFN treatment for 6 h in HCT116 cells. **E** Immunoblot analysis of BAZ1A protein expression in HCT116 cells treated with SFN or 6-SFN for 24 h. Differences between means for n = 3 replicates are indicated by ***p < 0.001 and ****p < 0.0001, using Student’s t-test vs. vehicle (DMSO).

Previous studies with SFN and its structural analog, 6-SFN, identified roles in deacetylase inhibition [23–27] and alternative RNA splicing [32]. In colon cancer cells treated as reported [26] with SFN or 6-SFN (Fig. 5A), primers designed to detect alternative splicing of *BAZ1A* around exon 13 (Fig. 5B) identified an increase in a 288-bp PCR product relative to the 384-bp product (Fig. 5C), indicative of exon exclusion. This was corroborated using qPCR and exon-specific primers to quantify the exon-skipped *BAZ1A-SF* transcript versus the full-length transcript *BAZ1A-LF* following treatment with SFN and 6-SFN (Fig. 5D). Sanger sequencing of the amplified PCR products confirmed that the alternatively spliced *BAZ1A* transcript underwent exon 13 exclusion (Fig. 5B), with immunoblotting revealing an increase in the BAZ1A-SF isoform following SFN and 6-SFN treatment compared to vehicle control (Fig. 5E). Interestingly, unlike SFN or 6-SFN, other established HDAC inhibitors did not generate BAZ1A-SF (Fig. S2).

**Fig. 6:**
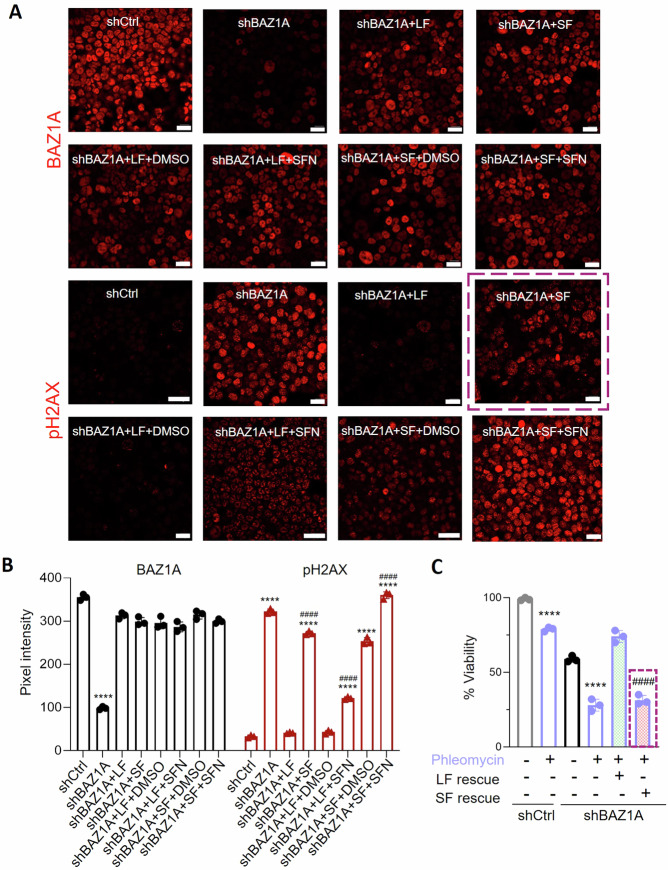
Loss of DNA repair activity in the presence of alternatively spliced BAZ1A enhances chemosensitization in colon cancer cells. **A** Immunofluorescence analysis of BAZ1A and pH2AX of HCT116 shBAZ1A KD cells transfected with BAZ1A-LF or BAZ1A-SF constructs, followed by SFN treatment for 6 h. **B** Quantification of pixel intensities from (**A**). **C** Cell viability assay comparing the chemosensitivity to phleomycin treatment (24 h) and rescue with BAZ1A-LF or BAZ1A-SF constructs in control (shCtrl) and BAZ1A KD (shBAZ1A) cells. Viability as a percentage relative to untreated shCtrl cells. Differences between means for n = 3 replicates were significant, as indicated; ****p < 0.0001, determined by one-way ANOVA compared to shCtrl; #### significant difference from the directly preceding treatment group (p < 0.0001), as determined by Student’s t-test.

We hypothesized that BAZ1A-LF is essential for correct DNA repair activity, and that BAZ1A-SF might undergo changes in conformation and tertiary structure that would interfere with DNA repair, thereby enhancing DNA damage and chemosensitivity to DNA-damaging agents, such as SFN [26]. Functional characterization confirmed that BAZ1A-LF rescued DNA damage markers in BAZ1A knockout cells, whereas BAZ1A-SF was less effective, especially following SFN treatment. Immunofluorescence imaging highlighted several key observations (Fig. 6A), including the following: (i) loss of BAZ1A protein expression was observed in BAZ1A knockout cells, as expected, and transient transfection of BAZ1A-LF or BAZ1A-SF constructs rescued BAZ1A to similar levels; (ii) SFN and vehicle treatment did not affect the re-established BAZ1A levels; *i.e*., DNA damage outcomes were not due to differential expression of the BAZ1A isoforms under the experimental conditions reported here; (iii) BAZ1A knockout cells had markedly increased pH2AX expression, indicative of DNA damage, and this was rescued by re-introduction of BAZ1A-LF but not BAZ1A-SF (Fig. 6A, dashed box), suggesting functional differences in DNA damage repair capacity between the two BAZ1A isoforms; (iv) the reduced ability of BAZ1A-SF to rescue DNA damage in BAZ1A knockout cells was further exacerbated by SFN treatment (Fig. 6A, lower right panel), as evidenced by increased pH2AX levels in the SFN-treated BAZ1A-SF group. Quantitative analyses confirmed the statistical significance of these findings (Fig. 6B), underlining the distinct roles of BAZ1A isoforms in cellular responses to DNA damage and the compounding influence of SFN treatment on these processes. Notably, BAZ1A knockout cells exhibited increased chemosensitivity to Phleomycin treatment, compared to the vector controls, and BAZ1A-LF re-introduction circumvented chemosensitivity to the DNA damaging agent, whereas BAZ1A-SF failed to confer such protective effects (Fig. 6C, dashed box). Collectively, these findings indicated that BAZ1A-SF lacked the intrinsic activity of the full-length protein in correcting for DNA damage, presenting a potential vulnerability that could be exploited therapeutically in combination with DNA damaging agents.

### DBIRD complex regulates *BAZ1A* alternative splicing

**Fig. 7:**
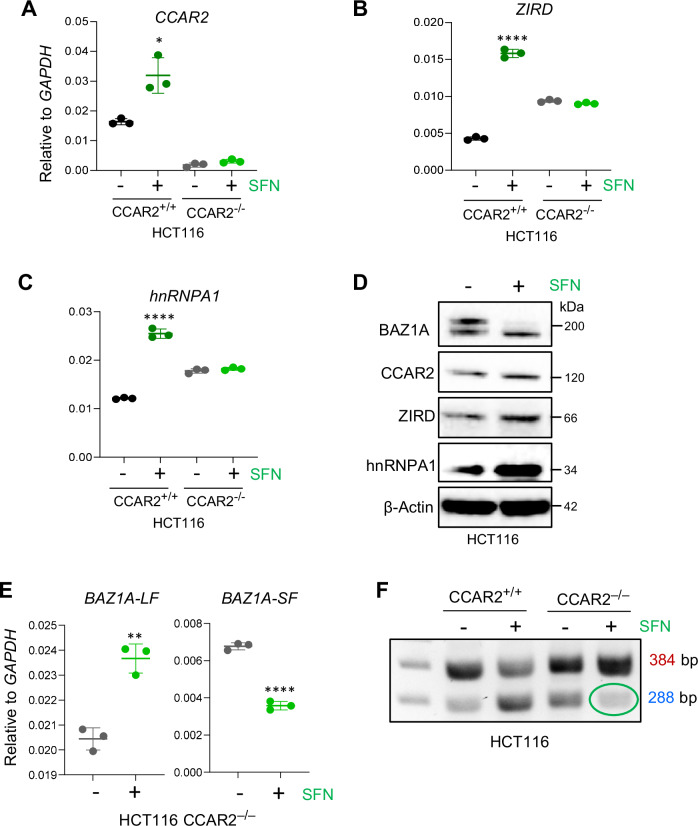
*BAZ1A* alternative splicing requires the DBIRD complex in SFN-treated colon cancer cells. **A**–**C** Quantitative RT-PCR analyses of DBIRD complex members [45] *CCAR2*, *ZIRD*, and *hnRNPA1* in HCT116 CCAR2^+/+^ and HCT116 CCAR2^−/−^ cells ±SFN treatment. **D** Immunoblot analysis in parental HCT116 cells treated with SFN for 24 h, with β-Actin as loading control. **E** RT-qPCR quantification of *BAZ1A*-LF and *BAZ1A*-SF transcripts upon SFN treatment for 6 h in CCAR2^−/−^ HCT116 cells. **F** PCR amplification of *BAZ1A* mRNA revealed reduced splicing in SFN-treated HCT116 CCAR2^−/−^ cells, highlighted by the almost complete loss of the 288-bp product (green circle). Statistical significance is denoted by *p < 0.05, **p < 0.01, and ****p < 0.0001.

Because BAZ1A is associated with CCAR2 in SFN-treated colon cancer cells [27], and CCAR2 is part of the DBIRD complex that regulates alternative mRNA splicing [45], the corresponding molecular targets were examined mechanistically. Incubation of parental HCT116 CCAR2^+/+^ cells with SFN increased the expression of DBIRD complex members (CCAR2, ZIRD, and hnRPA1) at the mRNA (Fig. 7A–C) and protein (Fig. 7D) level compared with vehicle controls, which was not observed in HCT116 CCAR2^–/–^ cells. Moreover, in CCAR2^-/-^ HCT116 cells, SFN treatment failed to generate BAZ1A-SF (Fig. 7E, F), demonstrating a critical requirement for CCAR2 in the *BAZ1A* splicing mechanism.

### In silico analysis implicated interference with the WAC domain in BAZ1A-SF

**Fig. 8:**
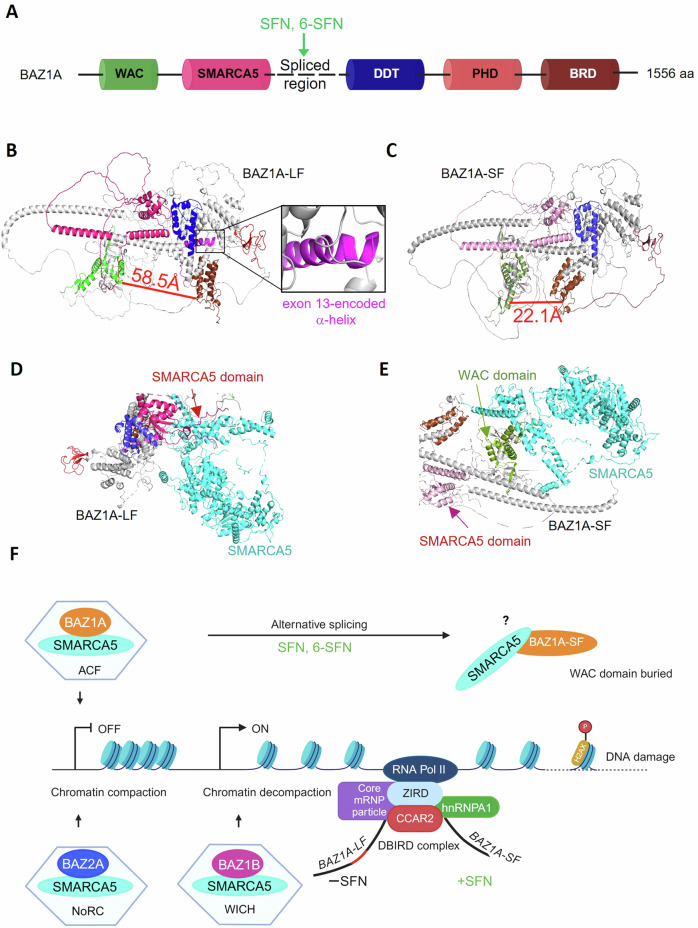
Structural implications of *BAZ1A* alternative splicing. **A** Domain architecture of the BAZ1A protein highlighting WAC, SMARCA5 interaction, DDT, PHD, and BRD domains, and the site targeted by SFN and 6-SFN for alternative splicing (arrow). **B** Three-dimensional structure of the BAZ1A long-form (BAZ1A-LF, Uniprot ID:Q9NRL2) with exon 13-encoded α-helix shown in detail, as analyzed by AlphaFold. **C** Structure of BAZ1A-SF (Uniprot ID: Q9NRL2-2), lacking the exon 13-encoded α-helix, as analyzed by AlphaFold v.2.3.0 and ColabFold.v.1.5.5. **D** SMARCA5 protein in close proximity to BAZ1A-LF SMARCA5 interaction domain (red arrow), as analyzed by HDOCKlite.v.1.1. **E** WAC domain of BAZ1A-SF buried within the SMARCA5 protein interaction site (green arrow), with distant SMARCA5 domain (red arrow). **F** Model for alternative splicing of *BAZ1A* and the involvement of the DBIRD complex. In SFN-treated colon cancer cells, the alternatively spliced short isoform of BAZ1A is predicted to undergo conformational changes that affect critical SMARCA5 interactions. The hypothetical inaccessibility of the DNA binding WAC domain in BAZ1A would interfere with DNA repair functions and enhance DNA damage in response to clinically used chemosensitization agents.

Full-length BAZ1A protein and the short isoform each contain WAC, SMARCA5, DDT, PHD, and BRD domains (Fig. 8A). Sequencing confirmed the involvement of exon 13 skipping and the loss of amino acid residues 504-535, which lie within a disorganized region between SMARCA5 and DDT domains (Fig. 8A). The full-length (BAZ1A-LF) protein structure was predicted by AlphaFold Protein Structure Database [46] via UniProt accession code Q9NRL2 (Fig. 8B). The alternatively spliced variant (BAZ1A-SF) was modeled by the standalone version of AlphaFold [47] using the FASTA file with UniProt sequence Q9NRL2-2 (Fig. 8C). Computational prediction revealed that BAZ1A-SF lacked an exon 13-encoded α-helix and loop region (Fig. 8C, expanded box). Notably, the absence of the α-helix in BAZ1A-SF reduced the interdomain distance between the WAC and BRD domains markedly, from 58.5 Å to 22.1 Å (Fig. 8B, C, red lines). *In-silico* docking with the interacting partner SMARCA5 was also examined. For the full-length BAZ1A-LF protein, the SMARCA5 interaction domain aligned close to the site of SMARCA5 protein contact (Fig. 8D, arrow). In marked contrast, the docked structure with BAZ1A-SF had the SMARCA5 interaction domain far removed (Fig. 8E, red arrow) and the WAC domain inaccessible within the SMARCA5 protein contact site (Fig. 8E, green arrow), making the WAC domain unavailable for linker DNA binding [48]. Docking scores for the SMARCA5/BAZ1A-LF and SMARCA5/BAZ1A-SF complexes were −236.0 and −251.2 kcal/mol, respectively, suggesting that BAZ1A-SF might compete for SMARCA5 interactions that destabilize the chromatin remodeling complexes due to the presence of a buried WAC domain (see working model in Fig. 8F). While these results are preliminary, and require experimental validation, they offer insights into the potential structural implications of BAZ1A-SF, particularly in terms of altered protein-protein interactions. The findings provide a basis for future experiments, including co-immunoprecipitation and nucleosome sliding assays with BAZ1A domain-specific mutants [18].

## Discussion

This investigation identified BAZ1A as a critical regulator of the DNA damage response, apoptosis, and senescence in human colon cancer cells, consistent with the diverse roles of BAZ family members and other chromatin remodeling factors in physiology and pathophysiology [4–12]. Interference with BAZ1A expression reduced the viability of colon cancer cells while increasing pH2AX, cleaved PARP, p16 and β-galactosidase activity. There was also a marked downregulation of the oncogenic Wnt/β-catenin signaling pathway, a gatekeeper of colorectal cancer progression due to *APC* or *CTNNB1* mutations that stabilize β-catenin [29–31, 49–51], with concurrent tumor growth inhibition in vivo compared to colon cancer cells that harbored constitutive BAZ1A expression.

The intriguing finding that SFN and 6-SFN caused alternative splicing of *BAZ1A* in colon cancer cells extended prior observations with SFN in a preclinical model of prostate cancer [32]. Since other established HDAC inhibitors did not generate *BAZ1A-SF* (Fig. S3), this implicates a role for non-acetylated proteins in *BAZ1A* alternative splicing. We postulated that the different isoforms of BAZ1A might exert competitive effects in colon cancer cells. Indeed, exon 13 exclusion produced BAZ1A-SF that was unable to circumvent DNA damage as effectively as the full-length BAZ1A-LF, with the short isoform maintaining chemosensitivity to phleomycin treatment. Computational modeling generated similar docking affinities with SMARCA5, suggesting competition between the two isoforms for SMARCA5 interactions. Notably, the altered conformation of BAZ1A-SF had the N-terminal WAC domain inaccessible for binding to nucleosomal linker DNA [18, 48]. This might provide a clue to the phenotypic outcomes in colon cancers following BAZ1A interference, disrupting the activity of ATP-utilizing chromatin assembly and remodeling factor (ACF) complexes (Fig. 8F). Mechanistic corroboration awaits further investigation.

The four members of the BAZ family exert diverse roles in the repair of DNA damage by ATP-dependent chromatin remodelers, altering DNA-histone interactions to mobilize, substitute or exclude nucleosomes [5, 18, 19, 48]. Recent advances have been made in our understanding of the different domains within the BAZ family and their functionality. For example, the PHD domain in BAZ1A binds to DNA, unlike that in BAZ1B, and the BAZ1A bromodomain interacts relatively weakly with acetylated histone peptides [18]. Nonetheless, BAZ1A and BAZ1B both recruit SMARCA5 to sites of damaged chromatin to promote survival, and their reader modules are critical for DNA damage recovery [18]. Given the significant role of SMARCA5 in BAZ1A-mediated DNA damage response, we also considered the potential involvement of SMARCA1. However, SMARCA1 was absent in the colon, according to protein expression data from the Human Protein Atlas (accessed 6/28/2024), underscoring the specific partnership between BAZ1A and SMARCA5 in colorectal cancer.

The focus of this work was on BAZ1A, but additional experiments were conducted to draw mechanistic insights into other BAZ family members in colorectal cancer. We observed that several of the phenotypic and molecular changes associated with BAZ1A interference were recapitulated for BAZ2A in colon cancer cells. This included (i) decreased viability and downregulation of Wnt/β-catenin signaling after BAZ2A genetic knockout (Fig. S4A–E), (ii) modest effects of the BAZ2-specific bromodomain inhibitor BAZ2-ICR [52] (Fig. S4F–I), and (iii) significantly reduced tumor growth for BAZ2A knockout cells in nude mice (Fig. S5A), with a concomitant reduction in Wnt/β-catenin signaling and histone protein expression in tumor xenografts (Fig. S5B and S5C). On the contrary, *BAZ1B* knockout increased cell viability (Fig. S6A–C), with a resultant decrease in cells occupying G_1_/G_0_ and an increase in S and G_2_M phases of the cell cycle (Fig. S6D). This was accompanied by increased Wnt/β-catenin signaling targets, such as c-Myc protein and mRNA expression (Fig. S6E, F), that was partially reversed by BAZ1B overexpression (Fig. S6G, H). These findings align with the diverse functions of BAZ family members [5, 18, 19, 48], and provide mechanistic insights for future precision oncology approaches based on the corresponding BAZ1A, BAZ1B, BAZ2A and BAZ2B molecular signatures, akin to targeting tumors with high β-catenin plus high CCAR2 expression for increased survival in colorectal cancer patients [29].

The DBIRD complex integrates alternative mRNA splicing with RNA polymerase II transcript elongation [45]. In colon cancer cells, SFN treatment increased expression of the DBIRD complex members (Fig. 7A–D) and demonstrated a requirement for CCAR2 in the BAZ1A splicing mechanism (Fig. 7E, F). A working model proposes that CCAR2 and other members of the DBIRD complex are activated upon SFN treatment, leading to *BAZ1A* alternative splicing (Fig. 8F). These observations would apply to other cancer types and their associated splicing factors [53–57], with the phosphorylation of CCAR2 by ATM/ATR signaling offering another level of regulation for BAZ1A splicing and function [28, 58]. Given the increased attention to RNA splicing deregulation in cancer etiology [59–61], additional mechanistic studies are warranted with pharmacological agents targeting specific BAZ family members, with a view to improved therapeutic interventions.

In conclusion, BAZ1A plays a crucial role in regulating the DNA damage response in colon cancer cells, and an alternatively spliced isoform was markedly less effective in DNA repair, enhancing chemosensitivity to DNA-damaging agents. In the clinical setting, patients with tumor-associated BAZ1A-SF might be more responsive to standard of care DNA-damaging agents, offering promising new avenues for precision oncology. Future research should focus on identifying specific BAZ family members that are deregulated in different cancer subtypes in order to enhance mechanistic targeting and improve upon current therapeutic interventions. Understanding the precise roles of BAZ1A and its isoforms could lead to more effective cancer treatments, thereby optimizing patient outcomes.

### Supplementary information

Supplementary Figures S1-S6 and Tables 1-2

Uncut Western blots

## Data Availability

All data pertaining to this study have been either presented in the paper or included in the Supplementary materials. Any resources generated during this research can be obtained upon request from the lead author, subject to the completion of a material transfer agreement.
