# Supplementary material for: Alternative splicing of BAZ1A in colorectal cancer disrupts the DNA damage response and increases chemosensitization
Source: Cell Death Dis. 2024 Aug 7;15(8):570. doi: 10.1038/s41419-024-06954-6 (PMC11306231; doi:10.1038/s41419-024-06954-6)
Supplement: Supplementary file 1 — Supplementary Figures S1-S6 and Tables 1-2 [file 41419_2024_6954_MOESM1_ESM.pdf]

Supplementary
Figures (S1-S6) and Tables (1-2)

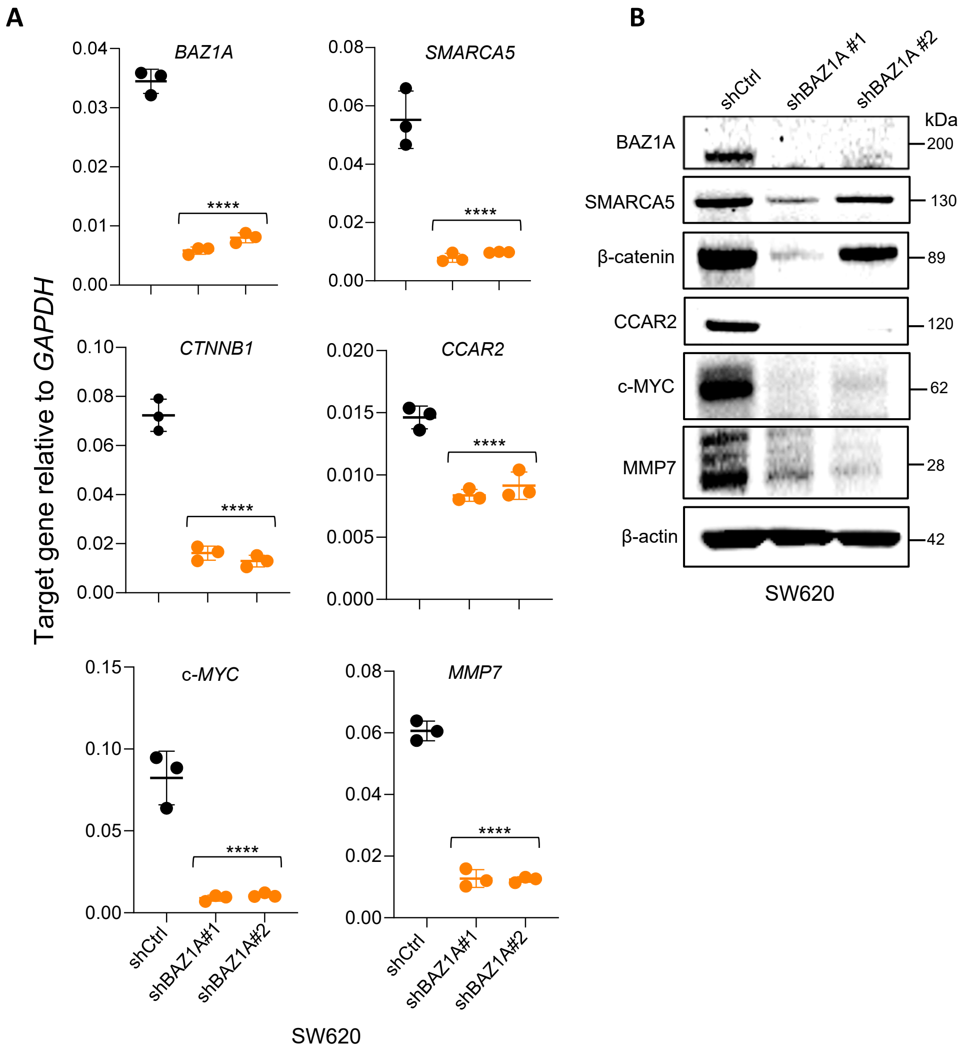

Supplementary Fig S1 BAZ1A knockdown downregulates the Wnt/β-catenin pathway in metastatic SW620 colon cancer cells. **A** qRT-PCR analysis of *BAZ1A*, *SMARCA5*, *CTNNB1*, *CCAR2*, c-*MYC*, and *MMP7* gene expression normalized to *GAPDH* in cells transfected with control shRNA (shCtrl) and two independent shRNAs targeting BAZ1A (shBAZ1A #1 and #2). **B** Immunoblotting of BAZ1A, SMARCA5, β-catenin, CCAR2, c-MYC and MMP7, with β-Actin as loading control. Statistical significance is denoted by ****$p < 0.0001$.

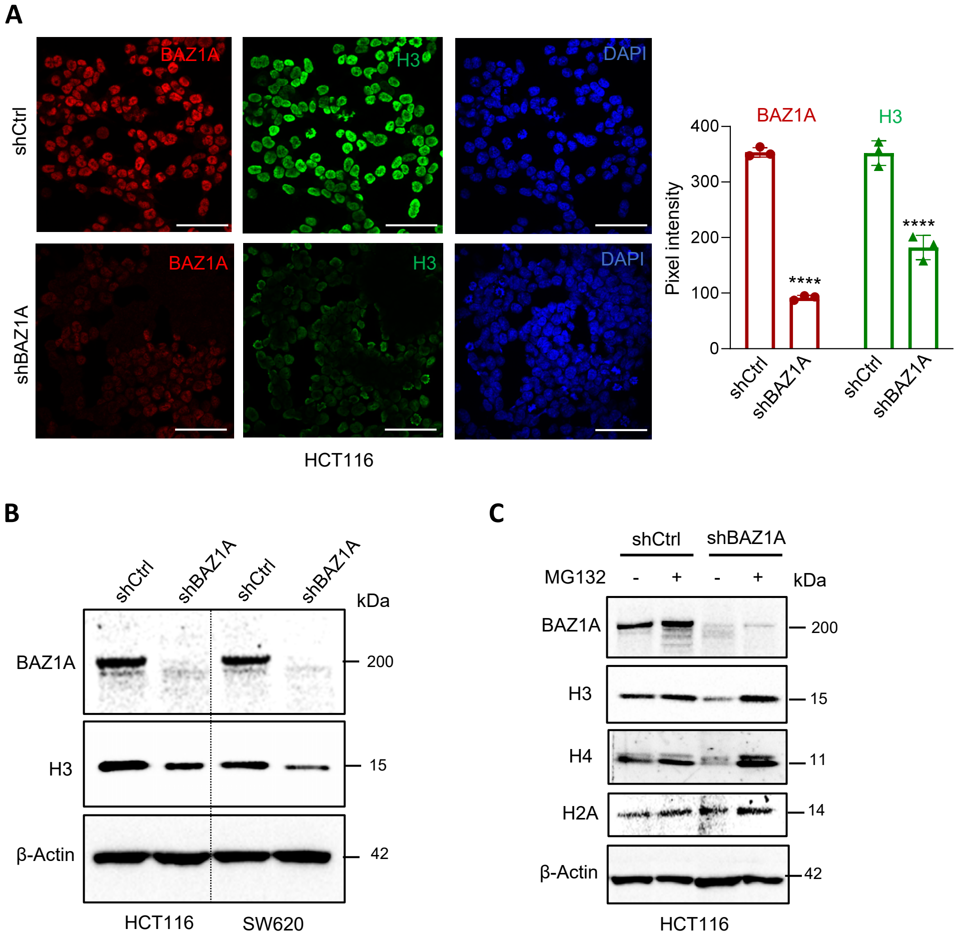

Supplementary Fig S2 BAZ1A knockdown reduces histone H3 and H4 protein expression in colon cancer cells. **A** Immunofluorescence analysis of BAZ1A (red), histone H3 (green), and DAPI (blue) in HCT116 cells transfected with control shRNA (shCtrl) or BAZ1A shRNA. Fluorescence intensity was quantified (right), with statistical significance denoted as ****$p < 0.0001$ by Student's t-test. **B,C** Immunoblotting of BAZ1A and histone proteins, with β-Actin as a loading control.

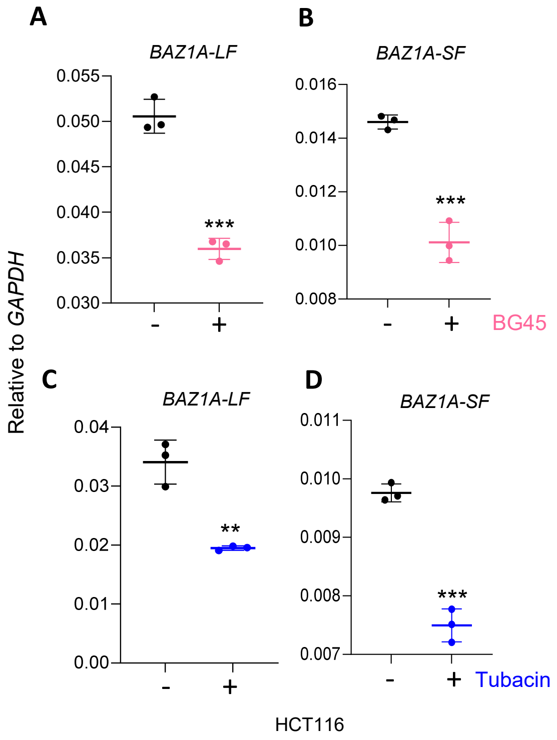

Supplementary Fig S3 HDAC inhibition is not synonymous with *BAZ1A* alternative splicing. Class I HDAC3-specific inhibitor BG45 (50μM) and class II HDAC6-specific inhibitor Tubacin (8μM) did not promote BAZ1A alternative splicing in colon cancer cells. RT-qPCR assays quantified for *BAZ1A* long form (LF) and short form (SF) transcripts after 6-h treatment with HDAC inhibitors. Differences between means for n = 3 replicates are indicated by **p , 0.01 and ***p < 0.001, using Student's t-test vs. vehicle (DMSO).

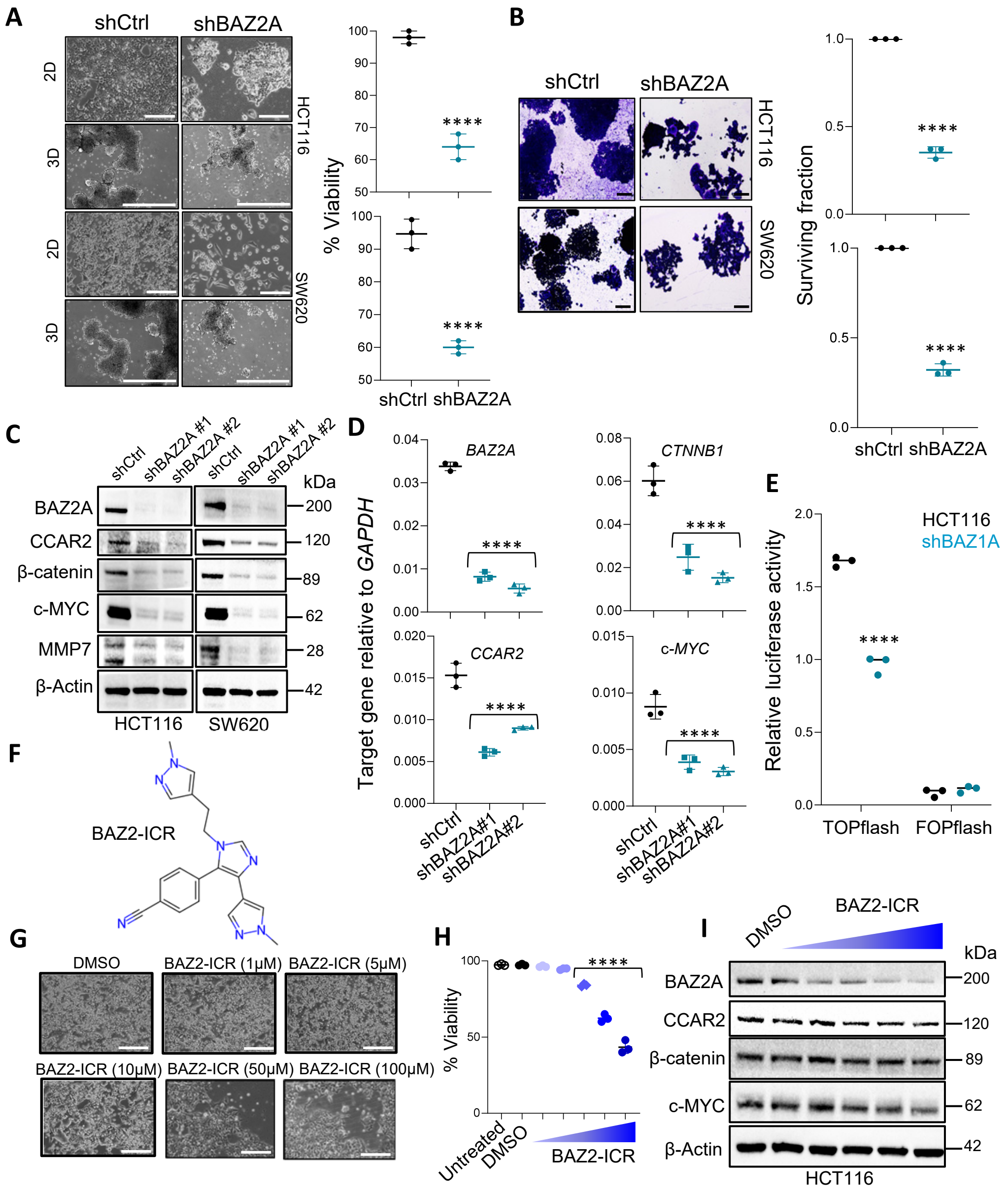

Supplementary Fig S4 BAZ2A knockdown and chemical inhibition in colon cancer cells. **A** Representative images and viability of HCT116 cells with control shRNA (shCtrl) and BAZ2A shRNA (shBAZ2A) in two-dimensional (2D) and three-dimensional (3D) cultures; scale bar = 200 μm. **B** Colony formation assay with quantitative analysis of surviving fraction. **C** Immunoblotting of BAZ2A, CCAR2, β-catenin, c-MYC and MMP7, with β-Actin as loading control. **D** qRT-PCR data of target genes normalized to *GAPDH*. **E** Luciferase reporter assay with TOPflash and FOPflash constructs indicative of Wnt/β-catenin activity. **F** Structure of BAZ2-ICR inhibitor. **G** Morphology of HCT116 cells treated with BAZ2-ICR for 5 days. **H** Cell viability in the CCK-8 assay 5 days after BAZ2-ICR treatment. **I** Immunoblotting 5 days after BAZ2-ICR treatment. Statistical differences between means from three replicates (****p < 0.0001) using Student's t-test or ANOVA vs. shCtrl or DMSO.

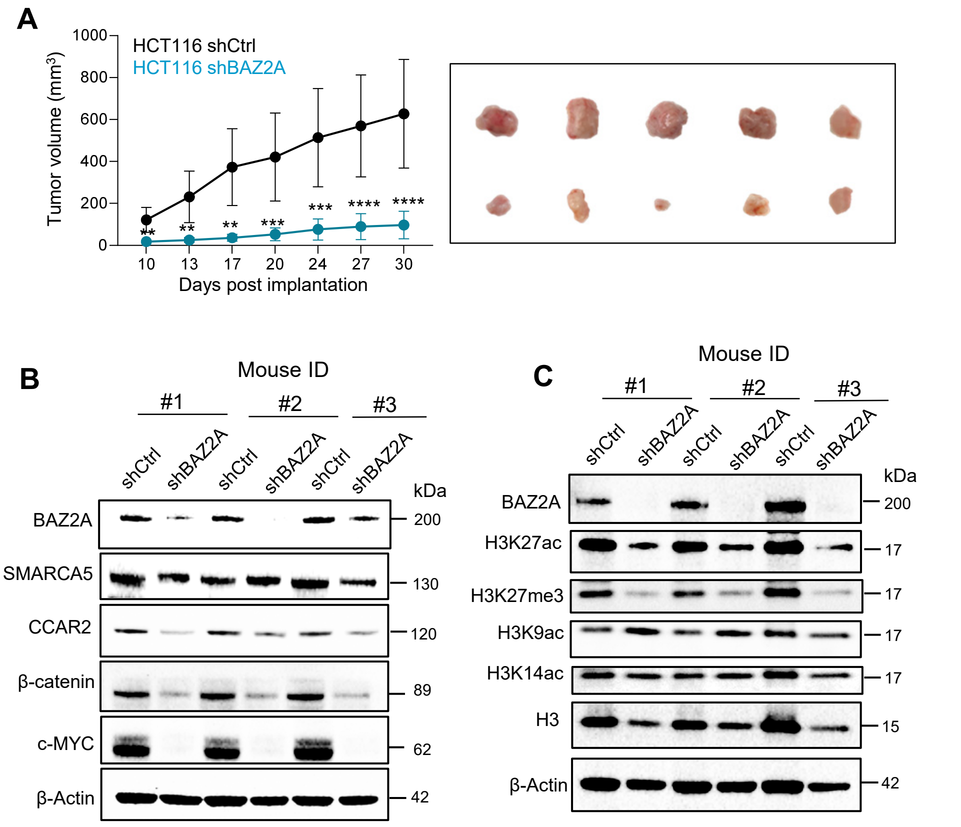

Supplementary Fig S5 BAZ2A knockdown in HCT116 tumor xenografts reduces tumor volume, downregulates Wnt/β-catenin pathway, and alters major histone modifications. **A** Tumor volume over time in nude mice implanted with HCT116 cells expressing control shRNA (shCtrl) or BAZ2A shRNA (shBAZ2A), with inset images showing harvested tumors at the end of the observation period. Each data-point represents mean±SD (n=5). **B&C** Immunoblotting in shBAZ2A or shCtrl xenografts with β-Actin as loading control. Statistical differences are indicated by $^{**}p < 0.01$, $^{***}p < 0.001$, and $^{****}p < 0.0001$ by Student's t-test vs. shCtrl.

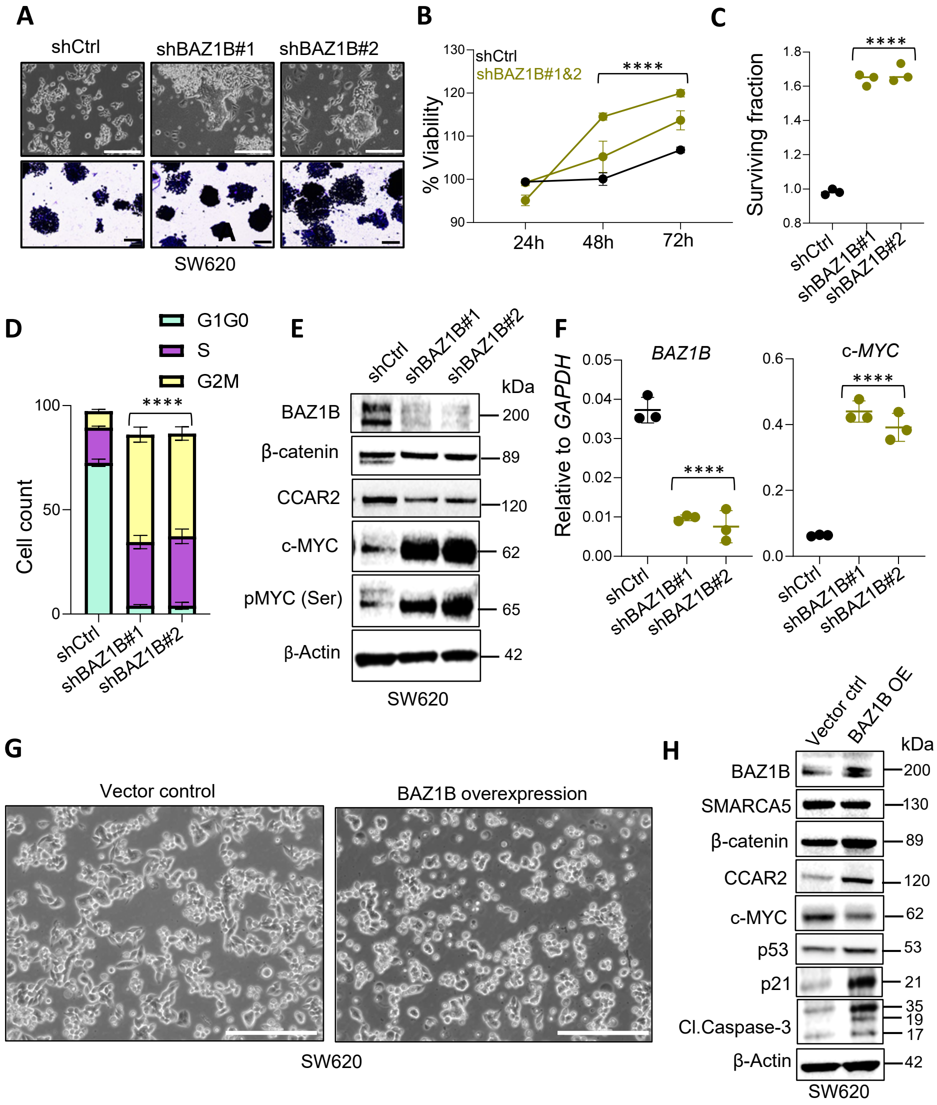

Supplementary Fig S6 BAZ1B knockdown and overexpression in SW620 cells. **A** Morphology and clonogenic assay of SW620 cells with control shRNA (shCtrl) and two independent shRNAs targeting BAZ1B (shBAZ1B#1 and #2). **B** Cell viability measured using CCK-8 assay at 24, 48, and 72h post-transfection. **C** Quantification of surviving fraction from clonogenicity assays. **D** Cell cycle analysis indicating the distribution of cells in G1/G0, S, and G2/M phases. **E** Immunoblotting of BAZ1B, β-catenin, CCAR2, c-MYC and phosphorylated c-MYC (Ser), with β-Actin as loading control. **F** RT-qPCR analysis of *BAZ1B* and *c-MYC* expression normalized to *GAPDH*. **G** Cell morphology upon BAZ1B overexpression compared to vector control. **H** Following BAZ1B overexpression (BAZ1B OE), SMARCA5, β-catenin, CCAR2, c-MYC, p53, p21 and Cleaved Caspase-3 proteins were immunoblotted, with β-Actin as loading control. Statistical significance is denoted by ****p < 0.0001.

Supplementary Table 1 List of shRNA target sequences. The table provides the sequences for shRNAs used to target various BAZ family genes.

shRNA	Target sequence
shBAZ1A#1	GCGATGAAGAAGAAGGTCAAA
shBAZ1A#2	CGACCGCATGTATAGACGATA
shBAZ1A#3	ATTGGATACAGTACTGGTTTA
shBAZ2A#1	CCTGCCTTTCAAGAAGGGATT
shBAZ2A#2	TATGCAACCTAGGCATCTTAA
shBAZ1B#1	GCCCTCTATGAAGTACCAGAT
shBAZ1B#2	CCCACAACAAATCTAGCTCTA

Supplementary Table 2 List of PCR primers. Forward and reverse primer sequences used for PCR amplification of target genes relevant to the study.

Target	Forward primer sequence (5'-3')	Reverse primer sequence (5'-3')
BAZ1A	CCTCTAGACAGAGACCATCCTT	CCTCCTCACTTTGACCTTCTTC
BAZ1A-LF	ACAGAGGCTTTGGATGAAGATG	CTGAGGTGCTTGACAGTTTCT
BAZ1A-SF	ACACCAAAGGCTGCAGTT	AGTTTCTTCACTAGACTGGGATTG
SMARCA5	CTCAGA AGACTCCAACTTCACC	CTGTTCTACGGTGTCGGTAATC
CTNNB1	ACTACCACAGCTCCTTCTCT	AAATCCCTGTTCCCACTCATAC
CCAR2	GATGATGGAGAGGAGGAGTTTG	GGCATGTCCTGAAGTGAAGA
c-*MYC*	AAGCTGAGGCACACAAAGA	GCTTGGACAGGTTAGGAGTAAA
MMP7	GTTAAACTCCCGCGTCATAGAA	GATCCTGTAGGTGACCACTTTG
ZIRD	CATGGACTTCGAGGACGATTAC	ACGATCCATCCCTCCATAAGA
hnRNPA1	GAAGGAGGCAGACAAGAGTAAG	CTTACCTACCCGCATAGCAATAA
BAZ2A	CCTACGACTGTCTCTGGAACTA	GATGCCGTTGAGTGGGTATT
BAZ1B	GCCTGTCATCCCAAGCTTAT	GCCCTCTTTGTCCCTTCATATT
GAPDH	GGTGTGAACCATGAGAAGTATGA	GAGTCCTTCCACGATACCAAAG
