# Supplementary material for: Alternative splicing of BAZ1A in colorectal cancer disrupts the DNA damage response and increases chemosensitization
Source: Cell Death Dis. 2024 Aug 7;15(8):570. doi: 10.1038/s41419-024-06954-6 (PMC11306231; doi:10.1038/s41419-024-06954-6)

Fig.1D

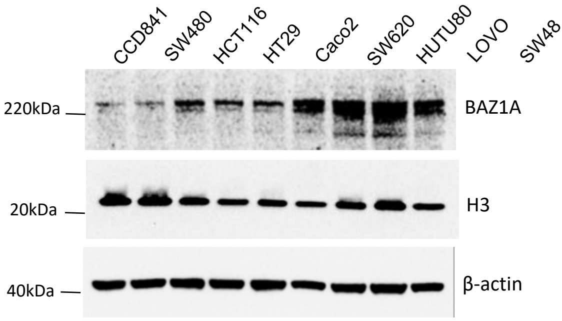
CCD841
SW480
HCT116
HT29
Caco2
SW620
HUTU80
LOVO
SW48
220kDa
BAZ1A
20kDa
H3
40kDa
β-actin

Fig.2A

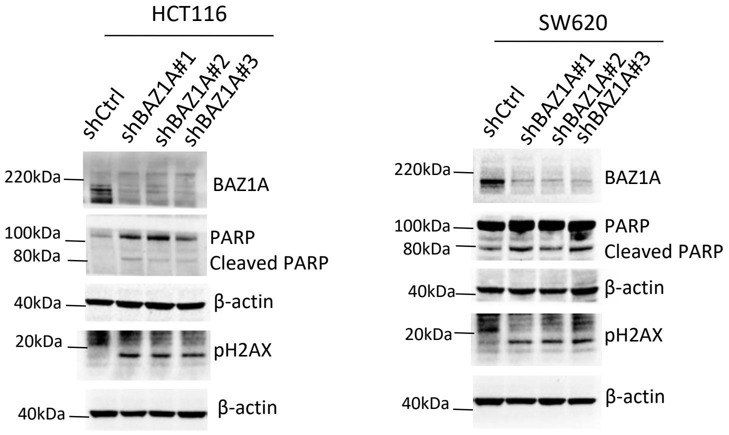
HCT116
shCtrl
shBAZ1A#1
shBAZ1A#2
shBAZ1A#3
220kDa
BAZ1A
100kDa
PARP
80kDa
Cleaved PARP
40kDa
β-actin
20kDa
pH2AX
40kDa
β-actin
SW620
shCtrl
shBAZ1A#1
shBAZ1A#2
shBAZ1A#3
220kDa
BAZ1A
100kDa
PARP
80kDa
Cleaved PARP
40kDa
β-actin
20kDa
pH2AX
40kDa
β-actin

Fig.2F

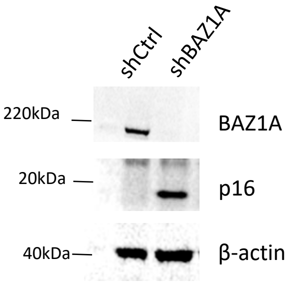
shCtrl
shBAZ1A
220kDa
BAZ1A
20kDa
p16
40kDa
β-actin

Fig.3A

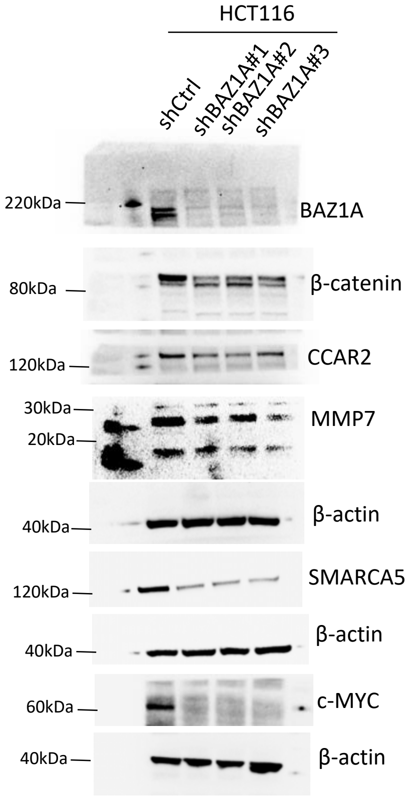
HCT116
shCtrl
shBAZ1A#1
shBAZ1A#2
shBAZ1A#3
220kDa
BAZ1A
80kDa
β-catenin
120kDa
CCAR2
30kDa
20kDa
MMP7
40kDa
β-actin
120kDa
SMARCA5
40kDa
β-actin
60kDa
c-MYC
40kDa
β-actin

Fig.4B

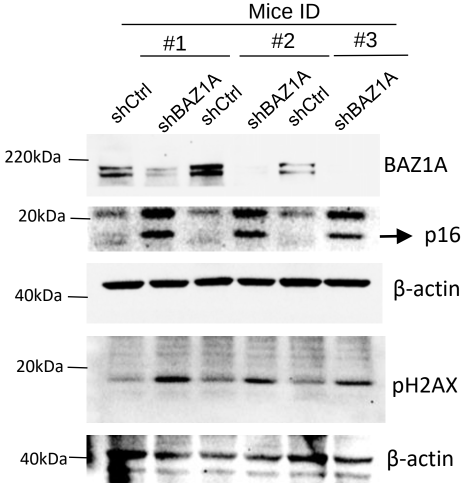
Mice ID
#1
#2
#3
shCtrl
shBAZ1A
shCtrl
shBAZ1A
shCtrl
shBAZ1A
220kDa
BAZ1A
20kDa
p16
40kDa
β-actin
20kDa
pH2AX
40kDa
β-actin

Fig.4C

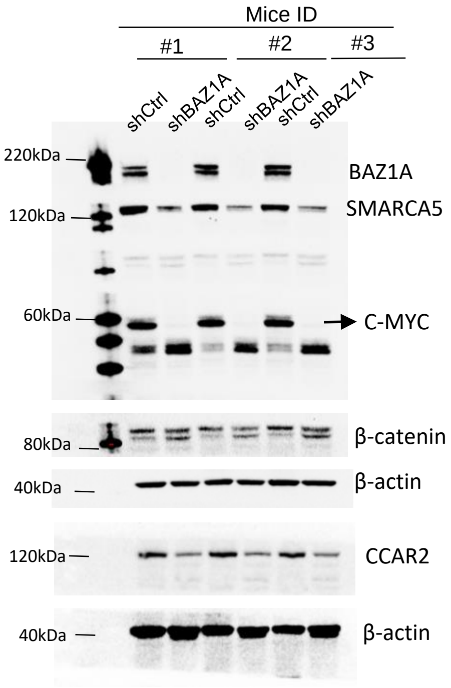
Mice ID
#1
#2
#3
shCtrl
shBAZ1A
shCtrl
shBAZ1A
shCtrl
shBAZ1A
220kDa
120kDa
60kDa
BAZ1A
SMARCA5
C-MYC
80kDa
β-catenin
40kDa
β-actin
120kDa
CCAR2
40kDa
β-actin

Fig.4D

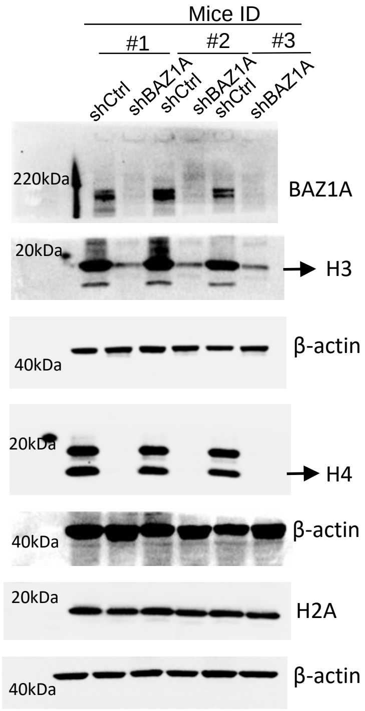
Mice ID
#1
#2
#3
shCtrl
shBAZ1A
shCtrl
shBAZ1A
shCtrl
shBAZ1A
220kDa
BAZ1A
20kDa
H3
β-actin
40kDa
20kDa
H4
40kDa
β-actin
20kDa
H2A
40kDa
β-actin

Fig.5E

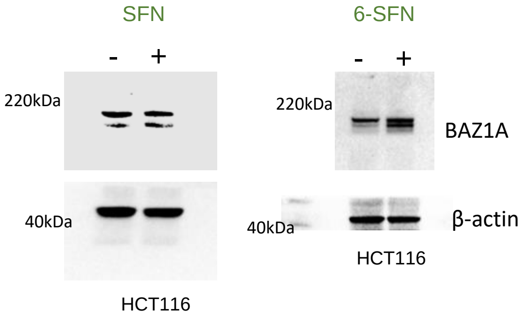
SFN
-
+
220kDa
40kDa
HCT116
6-SFN
-
+
220kDa
BAZ1A
40kDa
β-actin
HCT116

Fig.7D

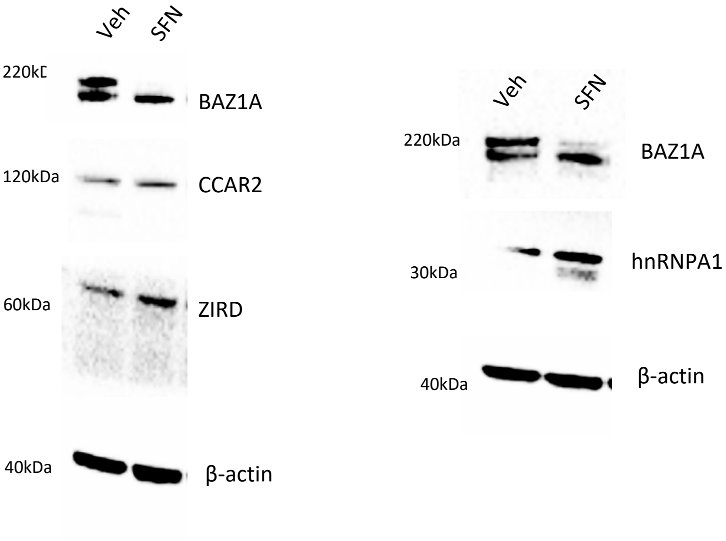
Veh
SFN
220kD
BAZ1A
120kDa
CCAR2
60kDa
ZIRD
40kDa
β-actin
Veh
SFN
220kDa
BAZ1A
30kDa
hnRNPA1
40kDa
β-actin

Fig.S1B

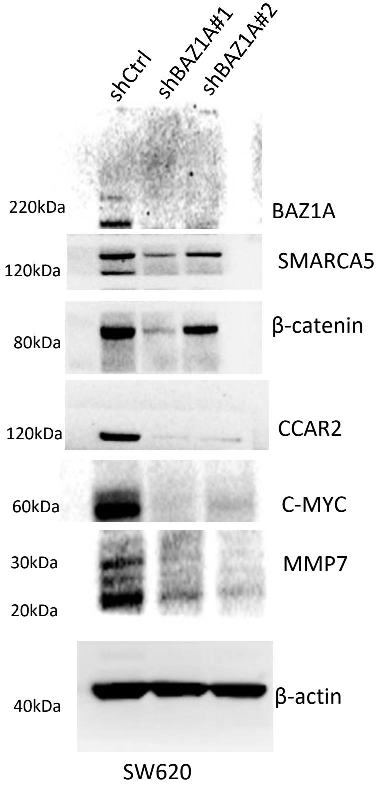
shCtrl
shBAZ1A#1
shBAZ1A#2
220kDa
BAZ1A
120kDa
SMARCA5
80kDa
β-catenin
120kDa
CCAR2
60kDa
C-MYC
30kDa
20kDa
MMP7
40kDa
β-actin
SW620

Fig.S2B

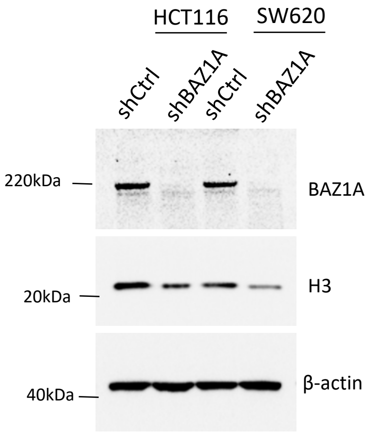

Fig.S2C

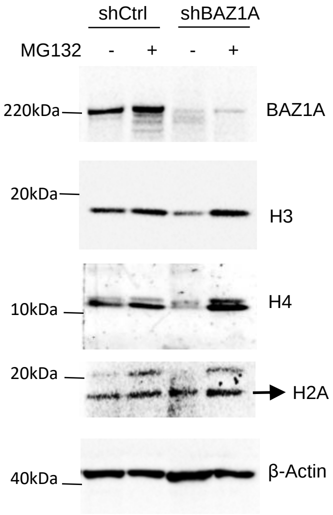

Fig.S4C

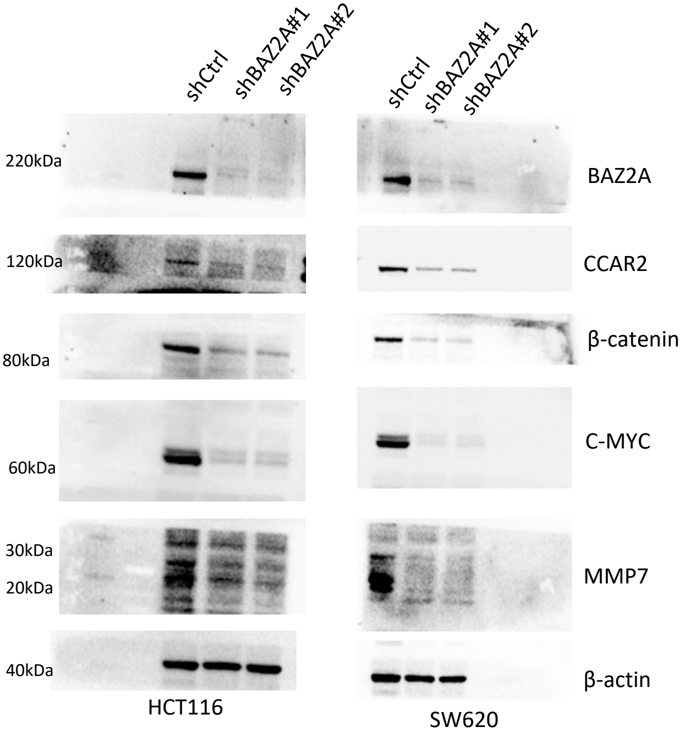
shCtrl
shBAZ2A#1
shBAZ2A#2
shCtrl
shBAZ2A#1
shBAZ2A#2
220kDa
120kDa
80kDa
60kDa
30kDa
20kDa
40kDa
BAZ2A
CCAR2
β-catenin
C-MYC
MMP7
β-actin
HCT116
SW620

Fig.S4I

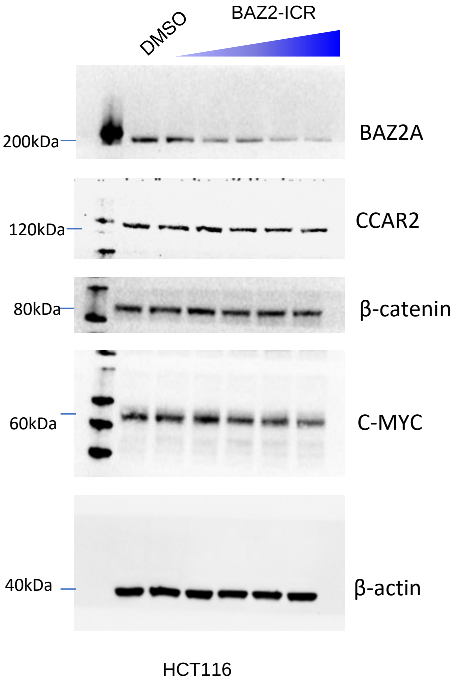

DMSO
BAZ2-ICR
200kDa
BAZ2A
120kDa
CCAR2
80kDa
β-catenin
60kDa
C-MYC
40kDa
β-actin
HCT116

Fig.S5B

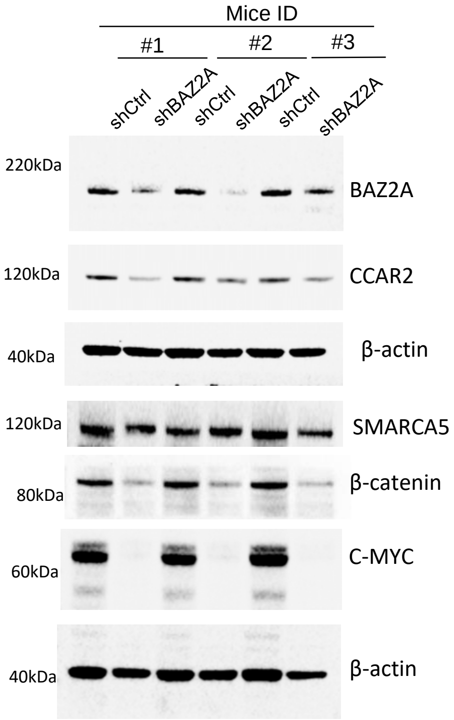
Mice ID
#1
#2
#3
shCtrl
shBAZ2A
shCtrl
shBAZ2A
shCtrl
shBAZ2A
220kDa
BAZ2A
120kDa
CCAR2
40kDa
β-actin
120kDa
SMARCA5
80kDa
β-catenin
60kDa
C-MYC
40kDa
β-actin

Fig.S5C

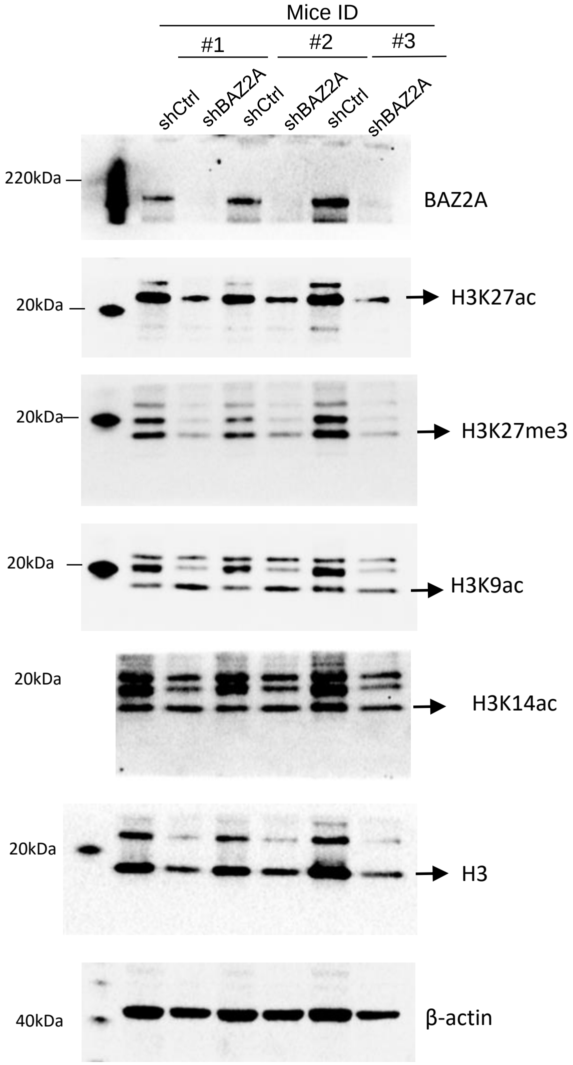
Mice ID
#1
#2
#3
shCtrl
shBAZ2A
shCtrl
shBAZ2A
shCtrl
shBAZ2A
220kDa
BAZ2A
20kDa
H3K27ac
20kDa
H3K27me3
20kDa
H3K9ac
20kDa
H3K14ac
20kDa
H3
40kDa
β-actin

Fig.S6E

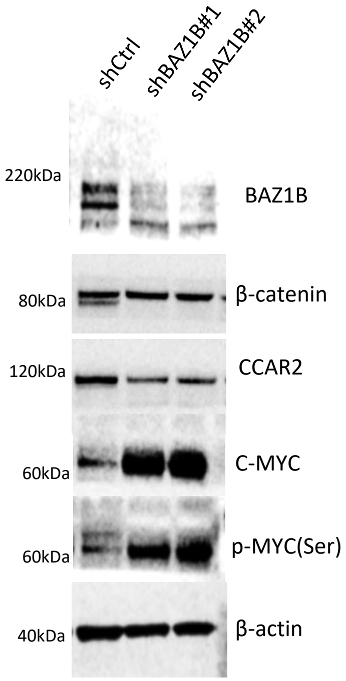

Fig.S6H

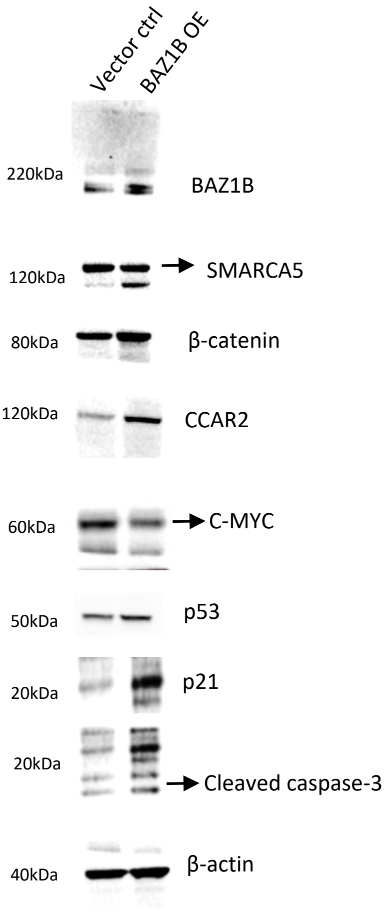

Supplement: Supplementary file 2 — Uncut Western blots [file 41419_2024_6954_MOESM2_ESM.pdf]
